# Formation of Microfiltration Membranes from PMP/PIB Blends: Effect of PIB Molecular Weight on Membrane Properties

**DOI:** 10.3390/membranes10010009

**Published:** 2020-01-03

**Authors:** Sergey Ilyin, Viktoria Ignatenko, Tatyana Anokhina, Danila Bakhtin, Anna Kostyuk, Evgenia Dmitrieva, Sergey Antonov, Alexey Volkov

**Affiliations:** A.V. Topchiev Institute of Petrochemical Synthesis, Russian Academy of Sciences, 29 Eninsky Prospect, 119991 Moscow, Russia; s.o.ilyin@gmail.com (S.I.); ignatenko@ips.ac.ru (V.I.); tsanokhina@ips.ac.ru (T.A.); Kostyuk@ips.ac.ru (A.K.); dmitrieva.ev.s@gmail.com (E.D.); antonov@ips.ac.ru (S.A.); avolkov@ips.ac.ru (A.V.)

**Keywords:** polyisobutylene, polymethylpentene, microfiltration membrane, extraction

## Abstract

A series of microfiltration membranes were fabricated by the extraction of polyisobutylene (PIB) from its immiscible blends with polymethylpentene (PMP). Three PIB with different molecular weight of 7.5 × 10^4^ (Oppanol B15), 34 × 10^4^ (Oppanol B50) and 110 × 10^4^ (Oppanol B100) g/mol, respectively, were used to evaluate the effect of molecular weight on the porous structure and transport properties of resulting PMP-based membranes. To mimic the conditions of 3D printing, the flat-sheet membranes were fabricated by means of melting of mixtures of various PMP and PIB concentrations through the hot rolls at 240 °C followed by a quick cooling. The rheology study of individual components and blends at 240 °C revealed that PIB B50 possessed the most close flow curve to the pure PMP, and their blends demonstrated the lowest viscosity comparing to the compositions made of PIB with other molecular weights (B15 or B100). SEM images of the cross-section PMP membranes after PIB extraction (PMP/PIB = 55/45) showed that the use of PIB B50 allowed obtaining the sponge-like porous structure, whereas the slit-shaped pores were found in the case of PIB B15 and PIB B100. Additionally, PMP/B50 blends demonstrated the optimum combinations of mechanical properties (σ_str_ = 9.1 MPa, *E* = 0.20 GPa), adhesion to steel (σ_adh_ = 0.8 kPa) and retention performance (*R*_240 nm_ = 99%, *R*_38 nm_ = 39%). The resulting membranes were non- or low-permeable for water if the concentration of PIB B50 in the initial blends was 40 wt.% or lower. The optimal filtration performance was observed in the case of PMP/B50 blends with a ratio of 55/45 (*P*_water_ = 1.9 kg/m^2^hbar*, R*_240 nm_ = 99%, *R*_38 nm_ = 39%) and 50/50 (*P*_water_ = 1100 kg/m^2^hbar*, R*_240 nm_ = 91%, *R*_38 nm_ = 36%).

## 1. Introduction

There are several ways to obtain porous polymer membranes: Phase separation [1,2,3,4,5], polymer crazing [6,7,8,9], bombarding by heavy ions [10,11], and extraction (or decomposition) of a component from the polymer matrix [12]. One of the varieties of the latter method is the use of a mixture of immiscible polymers: When one of the polymers serves as the basis of the membrane material and the second polymer is a temporary component of the mixture removed later by extraction to form a porous structure. This approach was used to obtain porous films from various polymer blends: Polypropylene and polybutene [13], polystyrene and polylactide [14], chitosan and poly(ethylene oxide) [15], chitosan and poly(vinyl pyrrolidone) [16], polyethylene and poly(ethylene oxide) [17], poly(4-methyl-1-pentene) (PMP) and polyisobutylene (PIB) [18,19], and polypropylene and polystyrene [20]. The preparation of membranes from a mixture of polymers is of particular interest since such a mixture can be applied in 3D printing of membrane precursors of complex form that can be used in compact membrane devices [21,22,23,24]. Not all polymers are well soluble, which makes it impossible to obtain membranes from them by phase separation of their solutions. Thus, the use of the melt of a polymer may be the only way to produce some product from it. Moreover, polymer solutions cannot be used in 3D printing. At the same time, the advantage of using additive technologies is the ability to manufacture small-size products with built-in membrane, which can be implemented on modern 3D printers with dual extrusion. 

One of the hardly soluble polymers is polymethylpentene. However, its poor solubility is also an advantage if one considers this polymer as a membrane material: Membranes based on it are chemically resistant. The main interest in microfiltration membranes based on PMP obtained by 3D printing may be related to obtaining small-sized smart devices capable of periodically taking samples of blood plasma and conducting their analysis. Firstly, due to the use of additive technology, a microfiltration membrane made of PMP can be built directly into the device case. Secondly, PMP is characterized by high hemocompatibility, e.g., this material is widely used to produce hollow fiber for blood oxygenators [25,26,27]. To use a blend based on this polymer to form a membrane precursor, a suitable second polymer is needed that can then be easily extracted. However, the question arises of how to control the morphology of polymer blends and transport properties of membranes based on them. In general, the morphology of a polymer blend is determined by the volume ratio of polymers, the ratio of their viscosities, and interfacial tension. The membrane polymer must be characterized by high chemical resistance and immiscibility with the second polymer, for example, due to crystallinity. In addition, the concentration of this polymer in the mixture should not be low and its viscosity should not be significantly lower than the viscosity of the second polymer during the mixing process, thereby allowing the formation of the continuous phase to the first polymer [28]. The second polymer should be soluble in a suitable solvent and provide a low interfacial tension in the mixture, which is crucial for the sizes of future pores.

The possibility of destruction and reduction of the size of the droplets of the disperse phase is determined by the capillary number:

(1) Ca = ηMγRζ

where *η*_M_, *R*, *γ*, and ζ denote the matrix viscosity, the droplet radius, the shear rate, and the interfacial tension, respectively. The capillary number should exceed a critical value, which depends on the ratio of component viscosities [29], and the optimal ratio of the viscosity of the disperse phase to that of continuous one is 0.1–1 [28]. The possibility of the polymer matrix viscosity increasing is limited, because high viscosity will complicate the mixing of the components and slow down the mixing rate, thereby reducing the capillary number. Thus, interfacial tension is the main parameter that determines the morphology of the polymer blend [30,31]. Since minimizing the interfacial tension is desirable, it is better to use another polyolefin for mixing with polymethylpentene. Polyisobutylene is the most soluble polyolefin that is easily dissolved in aliphatic hydrocarbons even at room temperature, while polymethylpentene is resistant to these liquids. Unfortunately, it is not always possible to select freely an extractable polymer or compatibilizer for reducing interfacial tension. In addition, despite the close chemical structures, the interfacial tension at the PMP/PIB interface is quite high: Approx. 7.8 mN/m [19]. Nevertheless, there is another possibility to influence the morphology of the mixture, which consists in using the viscous properties of the disperse phase, which usually fall outside the scope of researchers.

The capillary number determines the possibility of stretching and breaking of the droplets during the deformation of the blend. In this case, considering the size of the droplets in the blend, we mean an equilibrium state. However, what if we use a blend with morphology corresponding to its non-equilibrium state that is formed in the process of mixing? In this condition, the droplets of the disperse phase are in the extended state [29,32] that can be fixed by the rapid cooling of the blend. The rate at which droplets stretch during flow and relax at rest is determined by the viscosity of the disperse phase that can be easily changed by choosing the molecular weight of the extracted polymer. Of course, one cannot expect from this membrane preparation method a radical reduction in the size of the retained particles in the retentate. Nevertheless, this can be a way to fine-tune transport characteristics of membranes, in particular, reducing the pore size while maintaining the total porosity.

In this paper, we studied immiscible polyisobutylene/polymethylpentene blends with various molecular weights of polyisobutylene in order to consider the effect of this polymer viscosity on the rheology of the blends as well as the morphology and transport characteristics of polymethylpentene membranes. First, we selected polyisobutylene with optimal viscous properties that provided the greatest stretching of its droplets and then tried to optimize the ratio of polymers in the blend to achieve the best membrane properties.

## 2. Materials and Methods

### 2.1. Materials and Membrane Formation

As a membrane base, poly(4-methyl-1-pentene) (Mitsui Chemicals TPX MX004, Tokyo, Japan) with MFI_5 kg, 260 °C_ = 25 g/10 min was used. Polyisobutylene was applied as an extractable second component of the polymer blend. Three different PIB grades were considered: Oppanol B15, Oppanol B50, and Oppanol B100 (BASF) with the weight-average molecular mass of 7.5 × 10^4^, 34 × 10^4^, and 110 × 10^4^ g/mol, respectively. PMP/PIB blends with the component ratio of 45/55, 50/50, 55/45, 60/40, 65/35, and 70/30 were prepared in a HAAKE Polydrive twin-rotor mixer equipped with sigma-blade rotors. The blends were mixed at 240 °C for an hour with a rotors speed of 30 rpm.

Membrane precursors in the form of PMP/PIB films with the thickness of 70 ± 10 μm were fabricated by placing polymer blends between two layers of a silicone-treated anti-adhesive polyimide film and pulling the thus-obtained three-layer film through the rollers of a ChemInstruments HLCL-1000 laminator at a temperature of 240 °C and laminating speed of 0.15 m/min (which corresponded to an average shear rate in the gap between the rolls of about 50 s^−1^). The films were cooled down to room temperature (since the melting point of the polymethylpentene is 233 °C, rapid crystallization of this polymer can be expected when it leaves the hot rolls) and then were immersed in heptane bath for 24 h to wash out PIB from PMP matrix. After that, the obtained membranes were placed in another heptane bath for 2 h and finally dried at ambient conditions. Weight of each polymer film was measured both before and after extraction of polyisobutylene. It turned out that all mass of polyisobutylene was extracted as a result of this procedure (within the limits of the weighing error). Film shrinkage because of PIB extraction did not exceed 5–10% by length and width.

### 2.2. Methods

Differential scanning calorimetry (DSC) (New Castle, DE, USA) was carried out by using a TA Instruments MDSC 2920 calorimeter within the temperature range from 50 to 270 °C at the heating and cooling rates of 10 °C/min in an argon medium. Then, 10 mg of test sample was placed in aluminum cups, which were then sealed but not pierced.

The flow curves of PIB/PMP blends were measured by a TA Instruments DHR-2 rotational rheometer at 240 °C using a cone–plate geometry (25 mm diameter, 2°) with a stepwise increase of shear rate from 0.01 to 100 s^−1^.

PMP/PIB blends adhesion to the steel surface was measured on a Stable Micro Systems Texture Analyzer TA.XT plus probe tack tester (Stable Micro Systems Ltd., Godalming, UK). Adhesion strength was measured using a cylindrical rod (Stable Micro Systems Ltd., Godalming, UK) with a diameter of 9.94 mm, a contact time of 60 s, a pressure of 12.6 kPa, and a rate of rod rise from polymer blend film of 0.1 mm/s.

Tensile tests of PMP membranes (60 mm length, 10 mm width) were performed with a ChemInstruments TT-1100 machine (ChemInstruments, Fairfield, OH, USA) at 25 °C with a constant speed of 3.8 cm/min. The maximum load of the sensor was 11.3 kg.

Membrane morphology was visualized by analysis of their cross-section area and surfaces with a Hitachi TM-3030Plus scanning electron microscope (SEM) (Hitachi Ltd., Tokyo, Japan). Acceleration voltage was equal to 15 keV. Vacuum at around 10^−6^ torr in the testing chamber was maintained by turbomolecular pump. To prepare membrane cleavages, the membranes were preliminarily impregnated in isopropanol and then broken in a liquid nitrogen medium. Using a Desk Sputter Coater (DSR-1) (Nanostructured Coatings Co., Tehran, Iran), the prepared samples were coated with a thin (5-nm-thick) layer of gold in a special chamber in a vacuum (about 50 torr).

The filtration experiments were carried out at room temperature and pressure of 1 atm in the setup with dead-end filtration cells (active service area was about 3.14 cm^2^) equipped with the stirring system. Polymethylpentene films were used as membranes after removal of polyisobutylene from them and without pre-compaction. The performance of membranes was characterized in terms of liquid permeability coefficient *P* (kg/m^2^hbar). Two systems with different particle sizes were used: One to assess the potential of films as microfiltration membranes and the other to evaluate their ability to ultrafiltrate. For microfiltration experiments, an aqueous dispersion of phthalocyanine particles, which was obtained with an IKA Ultra Turrax T18 disperser, with an effective diameter of 240 nm (according to dynamic light scattering on a Malvern Zetasizer Nano ZS analyzer (Malvern, UK) and a concentration of 0.01 wt.% was used. In addition, an aqueous solution of blue dextran (100 mg/L, the molecular weight of 500 kDa, and hydrodynamic diameter of 38 nm) was applied for ultrafiltration tests. The solute concentration in the feed and permeate was determined using a UV-VIS spectrometer PE-5400UV (Malvern Panalytical Ltd., Malvern, UK) at a wavelength of 600 nm and 620 nm for phthalocyanine and blue dextran, respectively. The retention coefficient was calculated as follows:

(2) $$R\left(\%\right)=\left(1-\frac{C_{p}}{C_{f}}\right)\cdot 100\%$$

where *C_p_* and *C_f_* are the solute concentrations in the permeate and the feed, respectively.

Single sample was used for calorimetry and rheology investigations (as they were characterized by reproducibility of experimental curves within a relative error of 5%), while 3–5 samples were tested for filtration, adhesion, and strength evaluations. Rheology, calorimetry, and adhesion were measured for PMP/PIB blends, while morphology, strength, and transport properties were investigated for polymethylpentene membranes.

## 3. Results and Discussion

### 3.1. Effect of PIB Molecular Weight

The condition for the droplets to stretch in the polymer blend is their presence, i.e., immiscibility of polymers at molding temperature. The miscibility of polymers improved with a decrease in their molecular weight, therefore there was a risk of PMP and PIB mutual solubility and suppression of crystallization of polymethylpentene while using a low-molecular-weight PIB.

It was found that the molecular weight of PIB practically did not affect the melting and crystallization of mixtures: The DSC curves of different blends with the same PIB content (45%) did not differ (Figure S1). Analysis of the curves showed a slight decrease in melting point of PMP with decreasing of PIB molecular weight (PIB B15, Table 1). This may indicate a slight solubility of PIB in PMP medium, which worsened with an increase of PIB molecular weight. Solubility slightly increased the crystallization temperature of the blend, which should have a positive effect on fixing the non-equilibrium film structure at the exit from the laminator rolls. In addition, one can note the unexpressed effect of PIB solubility on the crystallinity of PMP: On the one hand, the reduced enthalpy of PMP melting increased, and on the other hand, the reduced enthalpy of crystallization decreased.

**Figure 1:**
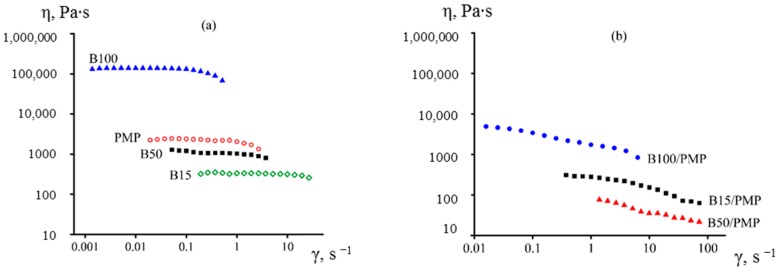
Flow curves of pure polymers (**a**) and PMP blends with 45% of PIB (**b**) at 240 °C.

Change in the molecular weight of PIB made it possible to vary its viscosity within three decimal orders. Thus, PIB viscosity can be higher, lower, or comparable with the viscosity of the PMP melt at the same temperature (Figure 1a). It was surprising that the viscosity of the blends was lower than the viscosity of both PMP and PIB, regardless of the molecular weight of the latter (Figure 1b). Thus, the small solubility of polyisobutylene in the polymethylpentene medium cannot cause a sharp decrease in viscosity, as the viscosity of solutions is to obey the rule of logarithmic additivity and cannot be lower than the viscosity of both components [33] (in addition, if the reason for the decrease in blend viscosity was the PIB solubility in PMP, the viscosity of the blend based on the least-viscous B15 would be lower than that of other blends). The low viscosity of the blends may be due to wall slip [34,35], which, however, is unlikely due to the very high adhesion of polyisobutylene to the steel surface (e.g., these polyisobutylenes are the basis of pressure-sensitive adhesives [36,37]). Another explanation may lie in interlayer slip [38,39,40] due to the low adhesion of polyisobutylene to PMP. A blend based on PIB with a moderate molecular weight has the lowest apparent viscosity, including in the range of shear rates used for film formation (~50 s^−1^). Based on this, the highest product output can be expected from PMP blends with this polymer. At the same time, the reason for the lowest apparent viscosity of this blend was unclear. The interfacial tension at the PIB/PMP boundary (and, therefore, the adhesion between the polymers) practically did not depend on the molecular weight, i.e., effect of interlayer slip should be the same. As for wall slip, the least apparent adhesion to the steel surface was exhibited by PIB with the highest molecular weight due to its higher viscosity that did not allow it to fill the roughness of the steel surface; however, the B100-based blend, on the contrary, had the highest apparent viscosity.

High adhesion properties of polyisobutylene led to the fact that the PMP/PIB blends were sticky to the touch. Moreover, a PMP blend with the lowest-molecular-weight PIB is characterized by rather high adhesion to steel (σ_adh_, Table 1). Adhesive strength of blend-to-steel bond was 5–15 times lower than that provided by conventional pressure-sensitive adhesives [41,42], but high enough to cause problems during storage and use of this blend, e.g., as a filament for 3D printing. The increase in the molecular weight of PIB led to a decrease in the stickiness of the blends, eliminating this problem.

**Figure 2:**
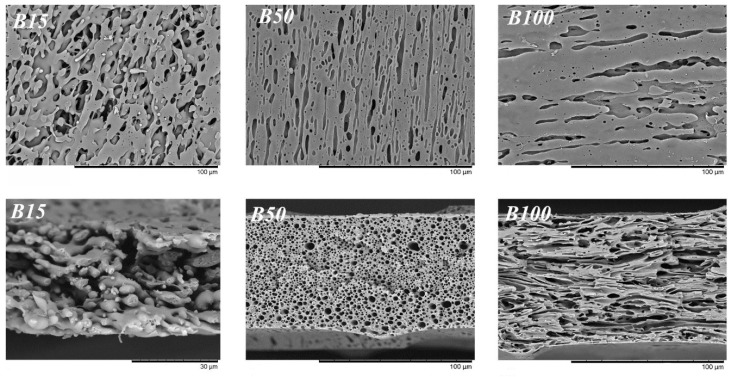
SEM images of surfaces (**top**) and cross-sections (**bottom**) of PMP membranes obtained from PMP/PIB blends with 45% of PIB. Membranes based on blends with B50 and B100 were broken across and along elongated pores, respectively.

Formation of a film from a PMP/PIB blend in a thin gap followed by rapid cooling and PIB extraction allowed analyzing the morphology of the membranes thus obtained (Figure 2). The surface of the film obtained from a PMP/B15 mixture was characterized by the presence of round holes. Thus, the use of this PIB did not lead to the formation of elongated pores, supposedly due to the rapid relaxation of stretched droplets of such low-viscous polymer. In other words, PIB droplets had time to lose their elongated shape after the film left the laminator rolls and before polymethylpentene matrix crystallization. Low strength of the membrane obtained from this blend can be expected since its cross-section was characterized by the presence of extensive interconnected cavities.

The use of B50 leads to the desired morphology of the membrane with long extended pores, while the pores of the membrane based on the PMP/B100 blend were also elongated, but had thickenings. Probably, B100 droplets did not have enough time to stretch while forming a film between the rollers of the laminator due to its high viscosity. Formation of threadlike fibrils in polymer blends was shown earlier for systems with interlayer slip [43], which was evidence in favor of the manifestation of this effect during viscosity measurements. Thereby, the effect of viscosity of polyisobutylene on the viscosity of its blends can be expressed in the fact that B50 formed the most elongated droplets, which provided better slip on the PMP/PIB interface and a greater reduction in the blend of apparent viscosity.

The strong elongation, thinning, and orientation of the pores of the membrane based on the PMP/B50 blend led to the best complex of membrane strength properties: Higher tensile strength, σ_str_, and Young’s modulus *E* (Table 1). The transport properties of the membranes were also improved (Table 2). A membrane obtained from the PMP/B50 blend had the highest retention factors for model contaminants (phthalocyanine *R*_240 nm_ and blue dextran *R*_38 nm_). Thus, the fundamental idea of this study was confirmed: The thinning of pores due to the elongation of disperse phase droplets in the membrane precursor during its formation provided an improvement in filtering properties. Unfortunately, this also reduced the permeate permeability through the membrane, probably due to the fact that the pores were elongated parallel to the membrane plane. Elongation of the pores parallel to the axis of mass transfer led to a significant increase in permeability while maintaining the same level of retention of model pollutants. However, some improvement in permeability can be achieved by increasing total porosity, which was easily achieved by increasing the concentration of polyisobutylene in the membrane precursor. In this case, the use of moderate molecular weight PIB (Oppanol B50) was most preferable: The blend on its basis was low viscous and nonsticky, while the resulting membrane had the most elongated pores, better barrier properties, and high strength. Investigation of PMP/PIB ratio influence on all of the above characteristics is of great interest.

### 3.2. Effect of PMP/PIB Ratio

**Figure 3:**
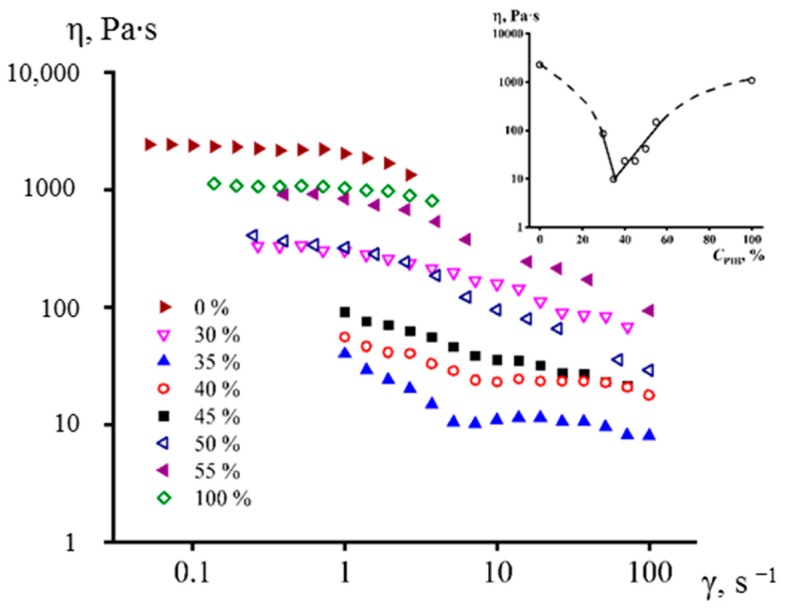
Flow curves of pure polymers and PMP/B50 blends with various content of B50 at 240 °C. The insert shows the concentration dependence of the apparent viscosity of PMP/PIB (B50) blends at a shear rate of 50 s^−1^.

A change in the PMP/PIB ratio did not significantly affect the thermograms of the blends (Figure S2). Regardless of the PIB concentration, all of them had comparable transition temperatures and the enthalpy of the PMP melting (Table S1). However, the ratio of polymers had a significant effect on the viscosity of the blends (Figure 3).

The viscosity of all the blends in the investigated concentration range was lower than the viscosity of both polymers, whose viscosities were comparable in magnitude. Thus, it is likely that all of these blends were characterized by interlayer slip. In addition, they were characterized by a decrease in apparent viscosity with an increase in shear rate. The capillary number grew with an increase in the shear rate (Equation (1)), which caused a greater elongation of the droplets and, most likely, an increase in the interlayer slip.

A PMP blend with 35% of PIB had the lowest apparent viscosity, which was 100 times lower than the viscosity of pure polymers (see the insert in Figure 3). From the point of view of morphology, such a mixture is characterized by smaller pore size and larger number of pores in comparison with mixtures that contain a larger amount of PIB. Moreover, a mixture with 50% of PIB has a fibrous structure. It can be explained by phase inversion: The fact that, at the given polymer ratio, the PIB became a continuous medium instead of PMP. Low PIB content led to the lack of pores in the center of a membrane, possibly due to the absence of pore percolation and incomplete PIB extraction.

**Figure 4:**
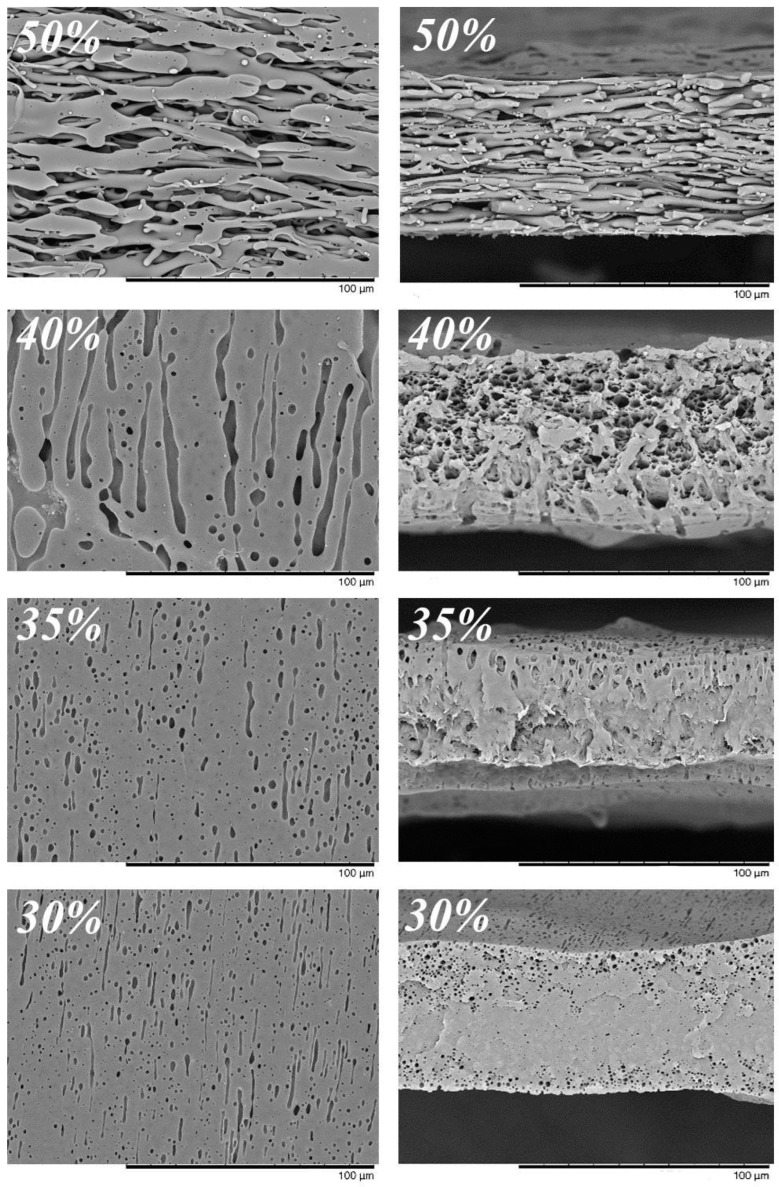
SEM images of surfaces (**left**) and cross-sections (**right**) of PMP membranes obtained from PMP/B50 blends with various content of PIB B50 that was indicated.

The difference in membrane morphology lead to a difference in their transport properties (Table 3). Membranes based on blends containing more than 40% of PIB were impermeable. The very low permeability of the membrane obtained from PMP blend with 40% of PIB (0.05 kg/m^2^hbar) disappeared after an attempt to filter contaminated water (with blue dextran or phthalocyanine particles), probably due to clogging of pores. An increase of the PIB content led to a significant increase in permeability but a gradual decrease in the retention coefficients of the model pollutants. In this case, the change in the concentration of polyisobutylene from 45 to 50% led to an abrupt increase in permeability of the resulting membrane, probably due to phase inversion in PMP/PIB blend, i.e., to the change of type of continuous medium from polymethylpentene to polyisobutylene (see Figure 2, middle, and Figure 4, top: In the first case, the pores have a stretched shape, while the polymer phase is elongated in the second one). In other words, the membrane turned out to be a sponge-like one if PIB content was 45%, while it became like a nonwoven fabric when PIB and PMP contents were equal. Such a fundamental transformation of the membrane structure resulted in a change in its permeability by almost three decimal orders.

Unfortunately, most of the membranes developed had very poor structural integrity, i.e., irregular voids. Such structure is never ideal for membranes, particularly for water filtration as it creates higher tortuosity that reduces water flux. At the same time, some membranes consisted of dense structure which is also not ideal for microfiltration. Nevertheless, the structure of membranes made from blends containing 45–50% of PIB B50 looked quite integral. The increase in permeability of membranes was associated with an increase in their porosity, which, however, reduced their strength properties (Table 3): The decrease in the strength and elastic modulus of the membranes occurred smoothly up to 55% PIB content. Further increasing of the PIB content in the mixture led to the formation of membranes with very low strength.

Despite the fact that many immiscible polymer blends were previously considered to produce porous films [13,14,15,16,17,18,19,20], for almost all of them, their transport properties were not measured. Membranes with water permeability of 96 and 200 kg/m^2^hbar were obtained from chitosan/poly (ethylene oxide) and polypropylene/polybutene blends, respectively [13,15]. However, their barrier properties were not measured. Thus, PMP/PIB blends allowed obtaining membranes with permeability exceeding by at least one decimal order than that of the others with similar history of preparation. As for commercially available microfiltration membranes, the chemically inert poly(tetrafluoroethylene-co-1,1-difluoroethylene) membrane (MFFK-1, STC Vladipor, Vladimir Russia) had comparable permeability with water (1310 kg/m^2^hbar) but worse barrier properties (*R*_240 nm_ = 58%).

## 4. Conclusions

In this work, a series of microfiltration membranes based on polymethylpentene (PMP) were fabricated. To this end, the immiscible blends of PMP with polyisobutylene (PIB) were prepared and then processed to flat-sheet films through the hot rolls at 240 °C (laminator) followed by a quick cooling. The porous structure of the resulting membranes was formed by selective extraction of PIB with heptane. To get insight in the role of PIB in the properties and performance of PMP/PIB blends and resulted PMP porous membranes, three PIB samples with different molecular weight of 7.5 × 10^4^ (Oppanol B15), 34 × 10^4^ (Oppanol B50), and 110 × 10^4^ (Oppanol B100) g/mol were taken. DSC revealed that all blends showed quite close melting and crystallization temperatures of PMP (*T*_m_ = 228.6–229.6 °C, *T*_cr_ = 203.8–205.4 °C), which was different from the neat PMP (*T*_m_ = 233.0 °C, *T*_cr_ = 202.9 °C). The maximum adhesion strength with steel surface (σ_adh_ = 10 kPa) was found for the blend based on PIB B15, while much lower adhesion was characteristic for PMP blends with PIB of 34 × 10^4^ (σ_adh_ = 0.8 kPa) and 110 × 10^4^ g/mol (σ_adh_ = 0.3 kPa) that simplified their processing. The rheology study of individual components and blends at 240 °C revealed that PIB B50 possessed the most-close flow curve to that of PMP, and their blend demonstrated the lowest viscosity comparing to the compositions made of PIB with other molecular weights (B15 or B100). SEM images of the cross-section of PMP membranes after PIB extraction (PMP/PIB = 55/45) showed that the use of PIB B50 allowed obtaining the sponge-like porous structure, whereas the slit-shaped pores were found in the case of PIB B15 and PIB B100. Additionally, blends based on PIB B50 demonstrated the optimum combinations of mechanical properties (σ_str_ = 9.1 MPa, *E* = 0.20 GPa), adhesion to steel (σ_adh_ = 0.8 kPa), and retention performance (*R*_240 nm_ = 99%, *R*_38 nm_ = 39%). The resulting membranes were non- or low-permeable for water if the concentration of PIB B50 in the initial blends was 40 wt.% or lower. The membranes made from PMP/PIB blends of 55/45 and 50/50 ratio demonstrated the optimal combination of water permeability and retention of two different solutes with the size of 38 and 240 nm. To sum up, immiscible blends of PMP with PIB B50 can be considered as a prospective system for fabrication of microfiltration membranes by melting processing that might include 3D printing techniques.

## Figures and Tables

**Table 1:** Temperatures *T* and enthalpies Δ*H* of melting and crystallization of PMP blends with 45% of PIB, their adhesion strength σ_adh_ with steel surface as well as strength σ_str_, and Young’s modulus *E* of membranes based on them.

PIB	*M*_PIB_, kDa	Melting			Crystallization			σ_adh_, kPa	σ_str,_ MPa	*E*, GPa
		*T*_m,_ °C	Δ*H*_m_, J/g	Δ*H*_m_/*C*_PMP_, J/g	*T*_cr_, °C	Δ*H*_cr_, J/g	Δ*H*_cr_/*C*_PMP_, J/g			
-	-	233.0	24.6	24.6	202.9	26.1	26.1	0	14.4	0.36
B15	75	228.6	16.6	30.3	205.4	11.7	21.3	10	5.1	0.17
B50	340	229.5	13.9	25.4	204.1	12.2	22.2	0.8	9.1	0.20
B100	1100	229.6	16.4	29.8	203.8	11.7	21.3	0.3	8.2	0.14

**Table 2:** Water permeability and retention of model contaminants for membranes based on PMP blends with 45% of PIB.

PIB	*P*_water_*,* kg/m^2^hbar	*R*_240 nm_, %	*R*_38 nm_, %
B15	31,000	93	8
B50	1.9	99	39
B100	76	58	3

**Table 3:** Mechanical (strength, σ_str_, and Young’s modulus *E*) and transport properties (water permeability *P*_water_ and retention coefficients *R*) of PMP membranes as a function of initial PIB B50 content in PMP/PIB blends.

*C*_PIB(B50)_, %	σ_str,_ MPa	*E*, GPa	*P*_water_*,* kg/m^2^hbar	*R*_240 nm_, %	*R*_38 nm_, %
0	14.4	0.36	non-permeable		
30	13.2	0.27			
35	11.7	0.22			
40	9.5	0.21	0.05	-	-
45	9.1	0.20	1.9	99	39
50	7.9	0.13	1100	91	36
55	3.3	0.09	3790	87	29

## Supplementary Materials

The following are available online at https://www.mdpi.com/2077-0375/10/1/9/s1. Figure S1: DCS curves of PIB/PMP blends: Heating (a) and cooling (b) scans at 10° C/min. Figure S2: DCS curves of B50/PMP blends with various B50 content: Heating (a) and cooling (b) scans at 10 °C/min. Table S1: Temperatures *T* and enthalpies Δ*H* of melting and crystallization of B50/PMP blends with various content of B50.
