# Supplementary material for: Formation of Microfiltration Membranes from PMP/PIB Blends: Effect of PIB Molecular Weight on Membrane Properties
Source: Membranes (Basel). 2020 Jan 3;10(1):9. doi: 10.3390/membranes10010009 (PMC7022575; doi:10.3390/membranes10010009)
Supplement: Supplementary file 1 [file membranes-10-00009-s001.pdf]

membranes

# Supplementary Materials: Formation of Microfiltration Membranes from PMP/PIB Blends: Effect of PIB Molecular Weight on Membrane Properties

**Sergey Ilyin, Viktoria Ignatenko, Tatyana Anokhina, Danila Bakhtin *, Anna Kostyuk, Evgenia Dmitrieva, Sergey Antonov and Alexey Volkov**

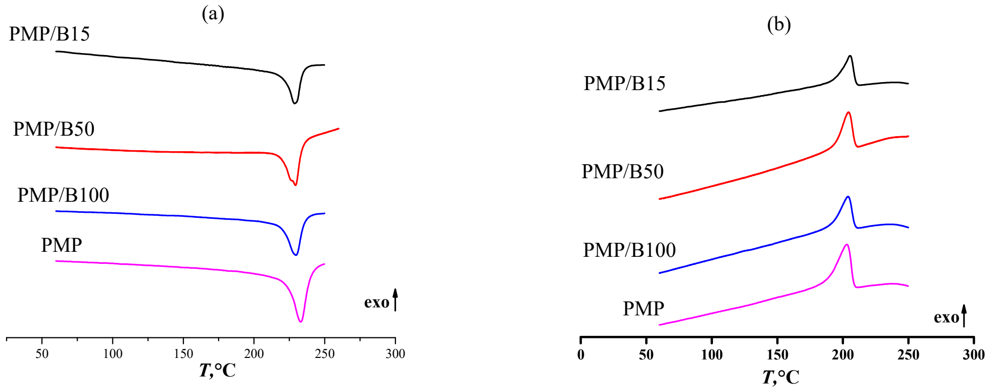


**Figure S1.** DCS curves of PIB/PMP blends: heating (**a**) and cooling (**b**) scans at 10°C/min.

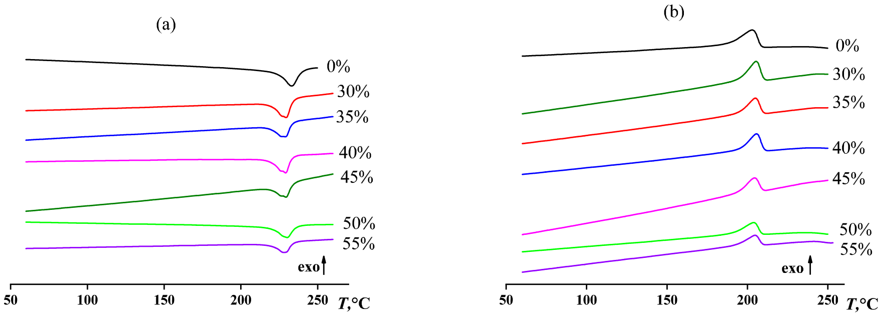


**Figure S2.** DCS curves of B50/PMP blends with various B50 content: heating (**a**) and cooling (**b**) scans at 10°C/min.

**Table S1.** Temperatures $T$ and enthalpies $\Delta H$ of melting and crystallization of B50/PMP blends with various content of B50.

| B50, % | Melting | | | Crystallization | | |
|---|---|---|---|---|---|---|
| | $T_m$, °C | $\Delta H_m$, J/g | $\Delta H_m/C_{PMP}$, J/g | $T_{cr}$, °C | $\Delta H_{cr}$, J/g | $\Delta H_{cr}/C_{PMP}$, J/g |
| 0 | 233.0 | 24.6 | 24.6 | 202.9 | 26.1 | 26.1 |
| 30 | 229.5 | 22.4 | 31.9 | 205.3 | 18.8 | 26.8 |
| 35 | 229.2 | 16.5 | 25.4 | 204.9 | 18.3 | 28.2 |
| 40 | 229.1 | 16.3 | 27.1 | 205.6 | 19.2 | 31.9 |
| 45 | 229.5 | 12.2 | 22.2 | 204.1 | 13.9 | 25.4 |
| 50 | 230.2 | 11.5 | 23.0 | 203.9 | 15.8 | 31.6 |
| 55 | 227.9 | 12.2 | 21.2 | 204.6 | 13.8 | 30.7 |
